# Progressive Thinning of Visual Cortex in Primary Open-Angle Glaucoma of Varying Severity

**DOI:** 10.1371/journal.pone.0121960

**Published:** 2015-03-27

**Authors:** Longhua Yu, Liqi Xie, Chao Dai, Bing Xie, Minglong Liang, Lu Zhao, Xuntao Yin, Jian Wang

**Affiliations:** 1 Department of Radiology, Southwest Hospital, Third Military Medical University, Chongqing, China; 2 Department of Radiology, 401st Hospital of the People’s Liberation Army, Qingdao, Shandong, China; 3 Ophthalmology research center, Southwest Hospital, Third Military Medical University, Chongqing, China; 4 McConnell Brain Imaging Centre, Montreal Neurological Institute, McGill University, Montreal, Quebec, Canada; Chinese Academy of Sciences, CHINA

## Abstract

The aim of this study was to investigate possible changes of cortical thickness in the visual cortex in primary open-angle glaucoma (POAG) of varying severity. Twenty normal controls (NC), 20 mild (MP) and 17 severe (SP) POAG patients were recruited and scanned using magnetic resonance imaging. Cortical thickness analyses with regions of interest (V1, V2, ventral V3, V4 and V5/MT+) were used to assess the cortical changes among the three groups. Furthermore, the associations of cortical thickness with retinal nerve fiber layer (RNFL) thickness and mean deviation of visual field were analyzed. Compared with the NC group, decreased cortical thickness was detected in the bilateral V5/MT+ areas in the MP group and the left V1, bilateral V2 and V5/MT+ areas in the SP group. Cortical thinning of the bilateral V2 areas was detected in the SP group compared with the MP group. In addition, cortical thinning of these visual areas was related to the ophthalmologic measurements. In conclusion, POAG patients exhibit cortical thinning in the bilateral V5/MT+ in the early stage of disease. The cortical degeneration in visual areas is discrepant with disease progressing and the dorsal pathway might be selectively damaged in POAG. Therefore, the cortical thinning of these visual areas may play a key role in the progression of POAG and can serve as a novel biomarker for accurately evaluating the severity of POAG.

## Introduction

Primary open-angle glaucoma (POAG) is a progressive optic neuropathy and is characterized by irreversible retinal ganglion cells (RGCs) and optic nerve fibers loss [1], resulting in corresponding psychophysical abnormalities, such as visual field loss. Although in the retina glaucoma selectively affects the layer of RGCs, primate experiments and human postmortem histologic studies have revealed that glaucomatous damage extends from the RGCs to the lateral geniculate nucleus (LGN) and even to the primary visual cortex (V1, striate cortex) [2–6]. These studies indicated that glaucoma is a complex disorder in which the whole visual pathway may be involved.

Recently, several voxel-based morphometry (VBM) studies on magnetic resonance imaging (MRI) data have examined the disruption of brain gray matter (GM) in patients with POAG. These studies have demonstrated GM atrophy in the V1 and the second major visual area (V2) [7–10], indicating that signs of neurodegeneration in visual cortex encompass upstream parts of the higher visual areas (extrastriate cortex). However, the VBM technique is limited by the fact that it conflates information about morphology, size and position [11]. Surface-based morphometry (SBM) is an alternative approach that enables independent measurements of cortical thickness across the continuous cortical surface [12,13], which may be likely to yield more precise information regarding the underlying disease mechanisms. More recently, cortical thinning [14,15] in BA17 (V1), BA18 (V2) and BA19 was shown in the POAG patients by using the SBM approach. Nevertheless, the majority of MRI-based studies focused on GM changes in the advanced/severe stage of POAG [7,9,10]. Because visual impairment from POAG could be irreversible, early detection is vital [16]. Our recent whole-brain studies [8,15] failed to detect the GM changes in the early stage of POAG, which might be attributed to the small sample size.

Additionally, the majority of the whole-brain VBM and SBM studies were only performed on the voxel/vertex and did not correspond to specific brain sulco-gyral structures or specific brain functional regions. That may cause difficulties or inconsistences when comparing the results with other similar studies. For example, the V5/MT+ (middle temporal gyrus), which plays a significant role in motion perception [17–19], has been reported to be decreased [8,15], increased [10], or unchanged [9] in the GM by the whole-brain cortical studies. Therefore, it is necessary to assess the changes of cortical thickness in different visual areas separately, which may lead to not only a better understanding of the disease progression but an update on biomarkers. Using the regions of interest (ROIs)-based approach, Boucard and colleagues [7] found that visual field defects caused by POAG were associated with reduction in GM density in the V1. In another ROIs study, Bogorodzki et al. [14] reported that the cortical thickness of the BA19 was significantly decreased in the left and right hemispheres. However, the two study either only evaluated the GM of the V1 or only focused on the end-stage of the POAG. The objective of the present study was to investigate possible alternations of the visual cortex in patients with varying degrees of POAG using the ROI-based cortical thickness analysis. A well-validated and highly accurate surface-based method [13] was used to examine cortical thickness of the V1, V2, V3, V4 and V5/MT+ areas. Additionally, the correlations between the cortical thickness in these visual areas and clinical measurements were also investigated.

## Materials and Methods

### Ethics Statement

This study was approved by the Human Research Ethics Committees of the Third Military Medical University and was conducted in accordance with the Declaration of Helsinki. Written informed consent was obtained from all of the participants.

### Subjects

Thirty-seven patients with first-diagnosed bilateral POAG were enrolled in this study. The following diagnostic criteria of the American Academy of Ophthalmology was used to identify POAG: intraocular pressure ≥ 21 mmHg, a glaucomatous optic disc and visual field abnormalities, and a nonoccludable anterior chamber angle without the characteristics of either congenital or secondary glaucoma. The exclusion criterion was a recorded diagnosis of any other ocular, neurological, or psychiatric disorder(s).

The normal control (NC) group was comprised of 20 age-, gender-, and education- matched healthy subjects. All the control subjects underwent a comprehensive ophthalmologic examination to exclude glaucoma and other eye diseases. All of the participants were right-handed and individuals with ophthalmic edema, inflammation or trauma, a history of amblyopia or ophthalmic surgery, age-related macular degeneration, or detectable brain abnormalities on MRI scans were excluded from the study.

### Retinal Nerve Fiber Layer (RNFL) Thickness and Visual Field Examination

RNFL thickness was measured quantitatively using Topcon 3D optical coherence tomography (OCT-1000, Topcon Corporation, Tokyo, Japan). Visual field examinations were performed using the 30–2 program of the Humphrey Visual Field Analyzer (Carl Zeiss Meditec, Dublin, Calif). The steps were described in detail in our previous article [15]. The universal RNFL thickness and mean deviation (MD) of visual field were recorded.

Because the visual inputs from the ipsilateral temporal hemiretina and the contralateral nasal hemiretina project to the ipsilateral hemisphere, the mean clinical measurements (RNFL thickness and MD of visual field) for both eyes [15,20] were also recorded for statistical analyses.

### Mild and Severe Patient Groups

Based on the Hodapp-Anderson-Parrish system [21], the patients were split into two groups (Table 1). The severe patient (SP) group (17 patients) was defined as MD scores < -6 dB in the better performing eye. This criterion includes the moderate, advanced and severe stages of the disease (stages 2–4). The mild patients (MP) group (20 patients) was defined as early stage disease (stage 1, -6 ≤ MD < 0 dB) in both eyes. Patients in either stage 0 (MD > 0 dB) or stage 5 (unable to perform Humphrey visual fields in the “worst eye”) were not enrolled in this study.

**Table 1:** Baseline characteristics of the subjects.

Characteristics	NC	MP	SP	*p* value
Age (years)	43.3 ± 15.1	43.6 ± 14.7	48.1 ± 16.6	0.59^a^
Male/female	11/9	11/9	12/5	0.55^b^
Education (years)	11.8 ± 4.2	11.9 ± 3.1	11.6 ± 3.7	0.98^a^
Cup-to-disc ratio				
Right eye	0.18 ± 0.06	0.58 ± 0.20	0.81 ± 0.10	< 0.001^c^
Left eye	0.19 ± 0.06	0.57 ± 0.19	0.79 ± 0.15	< 0.001^c^
Visual field MD (dB)				
Right eye	0.44 ± 0.65	-2.43 ± 1.52	-19.4 ± 8.53	< 0.001^c^
Left eye	0.40 ± 0.49	-2.66 ± 1.70	-23.7 ± 7.24	< 0.001^c^
mean bilateral eyes	0.42 ± 0.54	-2.55 ± 1.34	-21.6 ± 6.21	< 0.001^c^
RNFL thickness (μm)				
Right eye	111.3 ± 6.7	82.2 ± 16.7	57.1 ± 15.7	< 0.001^c^
Left eye	109.7 ± 7.2	83.5 ± 10.4	52.1 ± 11.4	< 0.001^c^
mean bilateral eyes	110.5 ± 6.7	82.9 ± 11.8	54.6 ± 12.1	< 0.001^c^

The data are expressed as the mean ± standard deviation. MD, mean deviation; NC, normal controls; MP, mild patients; SP, severe patients.

^a, b^ The *p* values for ANOVA and chi-square tests in the 3 groups, respectively.

^c^ The *p* values for the t tests between the MP and SP group.

### Structural MRI Acquisition

The MRI experiment was performed using a 3-Tesla scanner (Magnetom Trio, Siemens, Erlangen, Germany) with an 8-channel phased-array head coil. The subjects were required to close their eyes and avoid any movement during the image acquisition. 3D high-resolution structural images were obtained using a T1-weighted magnetization prepared rapid acquisition gradient echo (MPRAGE) sequence. A sagittal continuous no-interval scan covering the whole brain was performed with the following parameters: repetition time, 1,830 ms; echo time, 4.43 ms; inversion time, 1,100 ms; flip angle, 9°; matrix, 256 × 256 mm^2^; 176 slices; and voxel size, 1.0 × 1.0 × 1.0 mm^3^.

### MRI Data Processing

**Fig 1:**
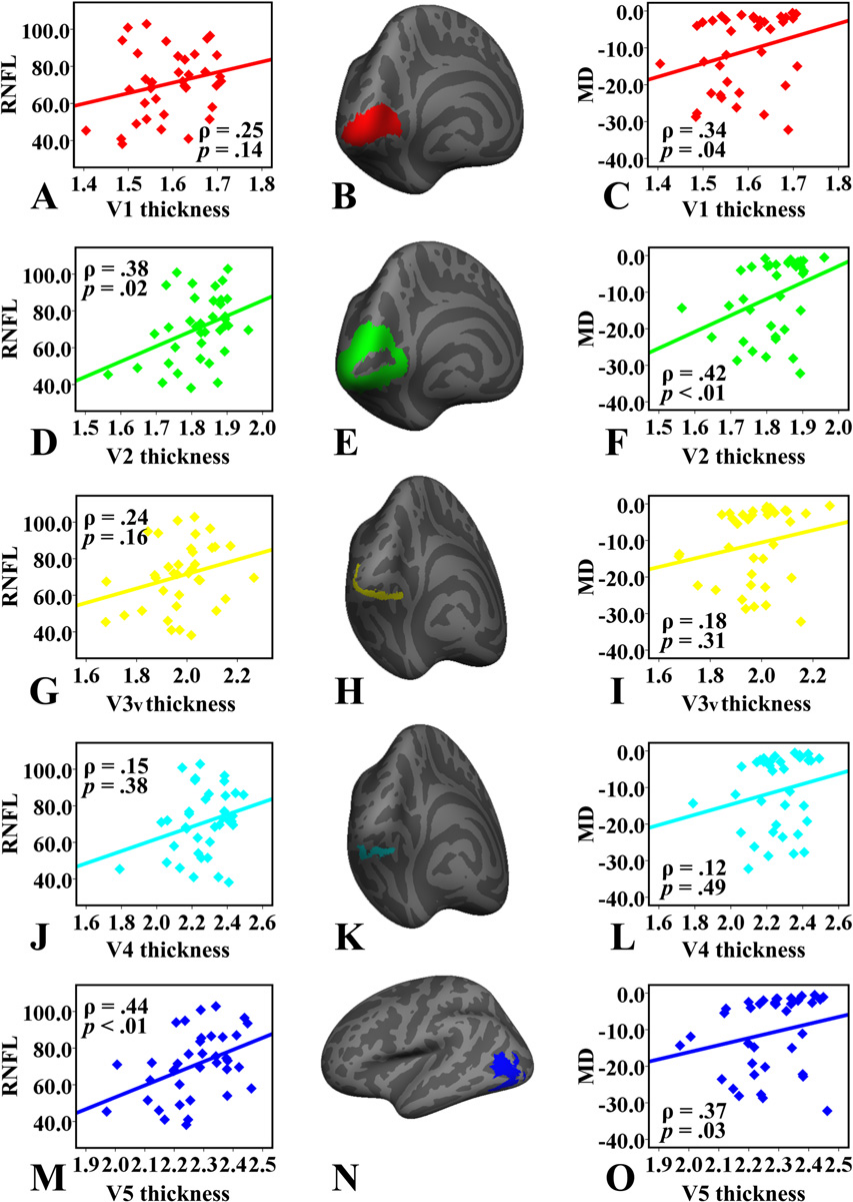
Correlations between cortical thickness of the visual areas and ophthalmic examination indexes. The labels of the V1 (B), V2 (E), V3v (H), V4 (K) and V5/MT+ (N) visual areas are only displayed on the left inflated surface. However, it should be noted that the average cortical thickness was computed for the homogeneous areas in bilateral hemispheres. RNFL, retinal nerve fiber layer thickness; MD, mean deviation of the visual field.

Cortical reconstruction and volumetric segmentation was performed with FreeSurfer (version 5.3.0, http://surfer.nmr.mgh.harvard.edu). The automated processing stream mainly included removal of non-brain tissue [22], Talairach transformation, segmentation of gray/white matter tissue [23], intensity normalization, topological correction of the cortical surface [24] and surface deformation to optimally place the tissue borders [25]. After creation of the cortical representations, the visual areas were parcellated based on existing atlases. The delineation of the V1 label was based on the study by Hinds et al. [26] and corresponded to Brodmann area (BA) 17 (Fig. 1B), and the V2 label was described by Fischl et al. [27] and corresponded to BA 18 (Fig. 1E). The delineation of the V5/MT+ label was based on the work of Malikovic et al. [28]. This V5/MT+ area was located close to the intersection of the anterior occipital and the inferior lateral occipital sulci in the region of the temporo-occipital junction (Fig. 1N). As the V3 and V4 templates are not available in FreeSurfer, we extracted the ventral V3 (V3v) and V4 using the Juelich histological atlas implemented in FSL (http://fsl.fmrib.ox.ac.uk/fsl/fslwiki/Atlases/Juelich). The templates were transformed into surface labels and manually corrected according to Rottschy’s description [29]. The V3v is buried deep in the collateral sulcus (Fig. 1H) and V4 is located on the lateral bank of this sulcus but also reaches the fusiform gyrus in the occipital section (Fig. 1K). The dorsal V3 was not delineated here because there are differing views regarding its anatomical location and additional subdivisions [30]. The labels were then registered to the surface of each subject using the spherical parameters of the cortical surface [31]. For each subject, the five visual areas were visually inspected for any inaccuracies in parcellation and manually corrected if necessary.

Cortical thickness was calculated as the average of the distance from the white matter surface to the closest point on the pial surface and from that point back to the closest point on the white matter surface [25]. For each visual area, we computed the average cortical thickness across that area.

### Statistical Analysis

Continuous variables were tested for normality using the Shapiro–Wilk test and visual inspection of histograms. Differences in the demographic and clinical measurements were assessed using analyses of variance (ANOVA), and the chi-square test was used for gender. Multiple analyses of covariance (MANCOVAs) were conducted to examine group differences in the cortical thickness results, using age and gender as covariates. A two-tailed *p* < 0.05 was considered statistically significant. Significant group effects were then further explored using Bonferroni post-hoc analyses (*p* < 0.05).

Finally, Spearman’s correlation analyses adjusted for age and gender were used to explore the associations between the mean clinical measurements (RNFL thickness and MD of visual field) for both eyes [15,21] and the mean cortical thickness of bilaterally homologous visual areas [32] across the whole patients. All of the statistical analyses were conducted using SPSS software (version 18.0, Chicago, USA).

## Results

### Demographic and Clinical Measurements

The demographic characteristics and clinical measurements are presented in Table 1. The three groups did not differ significantly with regard to age, gender or education. The clinical measurements of the SP group were significantly lower than those of the MP group (*p* < 0.001).

### Cortical Thickness

Table 2 shows the Bonferroni-adjusted differences in cortical thickness among the three groups. Compared with the NC group, the SP group had significantly lower thickness in the left V1 area and bilateral V2 and V5/MT+ visual cortices, whereas the MP group only exhibited a significant reduction of cortical thickness in the V5/MT+ area. Although the thickness of these visual cortices was reduced in the SP group relative to the MP group, only the differences in the bilateral V2 regions were statistically significant. In contrast, there were no significant differences in cortical thickness of V3v and V4 among the three groups.

**Table 2:** Comparison of cortical thickness among the NC, MP and SP groups.

Regions	Hemisphere	Cortical thickness (mm), Mean (SD)			*p* value			
		NC	MP	SP	ANCOVA	NC/MP	NC/SP	MP/SP
V1	left	1.63±0.11	1.58±0.07	1.55±0.07	**0.033**	0.320	**0.019**	0.829
	right	1.65±0.10	1.64±0.08	1.58±0.10	0.081	1.000	0.135	0.212
V2	left	1.86±0.11	1.82±0.06	1.74±0.09	**0.001**	0.600	**0.001**	**0.024**
	right	1.90±0.11	1.89±0.06	1.81±0.10	**0.003**	1.000	**0.012**	**0.047**
V3v	left	2.02±0.14	1.95±0.12	1.92±0.14	0.140	0.370	0.170	0.899
	right	2.07±0.16	2.08±0.12	1.99±0.15	0.210	1.000	0.384	0.327
V4	left	2.18±0.27	2.23±0.20	2.13±0.21	0.574	1.000	1.000	0.929
	right	2.44±0.20	2.36±0.10	2.33±0.20	0.341	0.634	0.605	1.000
V5/MT+	left	2.41±0.10	2.32±0.12	2.27±0.16	**0.013**	**0.037**	**0.005**	0.606
	right	2.38±0.16	2.28±0.11	2.20±0.14	**0.003**	**0.036**	**0.001**	0.177

NC, normal controls; MP, mild patients; SP, severe patients; *p* values corrected for multiple comparisons using the Bonferroni correction, *p* < 0.05. Bold values indicate statistically significant *p* values.

### Correlations between Cortical Thickness and Clinical Measurements

The Spearman’s correlation analyses showed that the mean RNFL thickness in the POAG patients was positively correlated with cortical thickness in the V2 (Spearman correlation coefficient ρ = 0.38, *p* = 0.02) and V5/MT+ (ρ = 0.44, *p* = 0.006) regions (Fig. 1D and 1M). Additionally, the mean MD was also related to cortical thickness in the V1 (ρ = 0.34, *p* = 0.04), V2 (ρ = 0.42, *p* = 0.009) and V5/MT+ (ρ = 0.37, *p* = 0.03) areas (Fig. 1C, 1F and 1O). No significant correlations was found between V3v/V4 cortical thickness and ophthalmologic measurements.

## Discussion

The present study examined the cortical thickness of the central visual areas in POAG patients. Compared with the NC group, decreased cortical thickness was detected in the bilateral V5/MT+ areas in the MP group and the left V1, bilateral V2 and V5/MT+ areas in the SP group. Additionally, cortical thinning of the bilateral V2 areas was detected in the SP group compared with the MP group. In addition, the thinning of visual cortex in POAG patients was significantly related to the deterioration of ophthalmologic measurements. These results indicated that the cortical thickness of specific visual areas could serve as biomarkers for the early diagnosis and judgment of progression in patients with POAG.

According to the cortical thickness analyses, cortical degeneration within the visual areas became progressively aggravated with the increasing severity of POAG. Consistent with the current findings, our recent vertex-based analysis across the whole brain revealed cortical thickness changes in the regions around the bilateral calcarine sulci [15]. In addition, several VBM studies have demonstrated a significant reduction in GM in the V1 and V2 areas [7–10], corresponding to the visual field defects caused by POAG [7,10]. However, the majority of these studies did not distinguish the differences of GM atrophy or functional defects among different stages of POAG, which is important to consider for understanding the progressive changes in the visual cortex. Besides, our previous studies also failed to pass the strict statistical correction for the multiple comparisons [8,15], which might be partly due to the heterogeneous changes of brain GM in POAG. In the present study, cortical thickness of the V1 and V2 areas did not change significantly in MP group but changed significantly in SP group, indicating that visual impairment is progressive and irreversible. Therefore, greater attention should be paid to prevention and early intervention for brain lesions in patients with POAG.

In POAG, the progressive reduction of thickness in the visual cortex may be attributed to the progressive loss of RGCs. Atrophy of the damaged parts of the retina likely propagates by means of transneuronal degeneration via the optic nerve towards the visual cortex, thereby provoking the subsequent degeneration of the visual cortex [33]. This sequence of events has been shown in both experimental animal models [34] and postmortem report [5]. In the present study, there was no significant alteration of the V1 area in the MP group, which may be explained by the fact that the majority of retinal inputs project to layer 4 of the V1 area. The two major visual pathways, magnocellular (M) and parvocellular (P) pathways, terminate primarily at layer 4Cα and layer 4A/4Cβ of the V1 cortex, respectively [35]. It is speculated that selective neurodegeneration may occur in layer 4 of the V1 area without extensively affecting the other five layers in the early stage of POAG. Because the six layers of the V1 area exist intracortical connections [36] and the death of neurons connected to the glaucomatous eye induces oxidative injury [37] and glial activation [34], the other five layers of the V1 area may be progressively disturbed as POAG worsens. Thus, cortical thickness in the V1 area exhibited a significant reduction in the advanced or severe stages of disease. Because the V2 area receives strong feedforward connections from the V1 area and has many properties in common with the V1 area, a similar mechanism of progressive neurodegeneration may also exist in the V2 area. Therefore, there was also no significant alteration in the cortical thickness of the V2 in the MP group.

The V2 degenerated rapidly while the V1 changed little when the disease deteriorated from mild to severe stage. A possibility may be that V2 neurons are modulated by more complex properties than V1, such as visual form [38,39] and motion processing [40,41], both of which are impaired in POAG patients [42,43]. Another possibility is that the topography in V2, which differs from the relatively orderly retinotopy of V1, is parcellated into functional modules which are locally retinotopic [44]. In addition, the size of V2 modules is greater than the orientation modules and blobs of V1, and the collection of hypercolumns for a point set spans the width of an entire stripe cycle in V2 (thin/pale/thick/pale) [44]. Taken together, more neurons and a larger area in V2 than V1 may be involved in the glaucomatous visual impairment with disease progression, and thus the cortical thickness of the V2 area was significantly reduced in the SP group.

Because visual impairment from POAG is aggravated progressively, early detection and diagnosis of glaucomatous disorders is vital [16]. In our previous studies, the mild patients (MP) group were defined as -10dB < MD < 0dB (Becker visual field stages) [8] or -12dB < MD < 0dB (including stages 1 and 2 in Hodapp-Anderson-Parrish system) [15] in both eyes. As more POAG patients were recruited, the MP group in the present study were redefined as -6 ≤ MD < 0 dB in both eyes, which is equal to stage 1 (early defect of visual field) in the Hodapp-Anderson-Parrish system [21], and the SP group were defined as MD scores < -6 dB (at least in stage 2) in the better performing eye. It is anticipated that the more stringent grading standard could help to reveal the earliest pathological features in POAG.

In the present study, we identified an alteration in cortical thickness of the V5/MT+ area in the early stage of POAG. The V5/MT+ area is located in the dorsal visual pathway and receives significant input from the M pathway [45]. Neuropathological examinations have revealed marked degenerative changes in the structures constituting this pathway, including RGCs [36], optic nerve fibers [46] and LGN [2–4]. In addition, neuronal activity in the V5/MT+ area play a key role in perception of visual motion [17,47]. Previous psychophysical studies have revealed disturbances in motion perception in patients with early POAG using either motion perimetry [48] or motion stimuli [49]. We therefore speculated that the degeneration of the V5/MT+ in early stage of disease might be interpreted as the absence of visual motion stimulation, as the absence of stimulation may result in changes in the cortical structure [50].

The pathology of glaucoma has been extensively studied at anterior visual pathway (M and P pathway) such as the retina and LGN. Most of these studies revealed a preferential damage to the M pathway [46,51,52] although a few of them supported a damage to the P pathway as well [2]. Recently, an autopsy study on a patient with advanced glaucoma found the degenerative changes in the brain involving the intracranial optic nerve, LGN and the V1 [5]. However, that study did not investigate the higher visual areas, such as the V3, V4 and V5/MT+ areas. The V3v and V4 areas locate in the ventral visual pathway, which mainly receive the visual inputs from the P pathway and play a significant role in processing color [53], form [54] and depth [55]. In this study, the cortical thickness in the V5/MT+ decreased significantly but there were no change in the V3v and V4 areas, indicating that the dorsal pathway may be selectively damaged in central visual system in POAG.

The significant positive correlations between cortical thickness of the visual areas and the ophthalmic measurements indicated that cortical thickness of the visual cortex in POAG patients varied consistently with RNFL and visual field damage. Previous studies have shown that diffusion parameters of optic nerves [56], optic tracts and optic radiations [21] correlated significantly with RNFL thickness in glaucoma. However, with regard to the visual cortex, the correlation between cortical thickness and the ophthalmic indexes has seldom been studied in human POAG. Our results provide new information about the neural basis of visual damage and support the use of cortical thickness analysis for studying patients with POAG. Thus, cortical thickness of visual areas may serve as a novel biomarker for determining POAG severity.

There are potential limitations that should be addressed. First, the sample size was relatively small, which reduces both statistical power and the ability to perform whole-brain analyses. Second, histological evidence of the observations cannot be provided because this study involved human subjects. As a result, our study could not verify whether layer 4 in the V1 is involved in the early stage of POAG. Future animal experiments and autopsies might resolve this problem. Third, because our study is cross-sectional in design, longitudinal studies should be employed to confirm our findings and to clarify the progression of the structural changes in visual areas in patients in the early stage of POAG. Finally, the current imaging study did not permit conclusions on the potential causal relationship between GM alternations in the visual cortex and ophthalmic lesions. Further investigations should be performed to explore the underlying molecular mechanisms.

## Conclusion

This study provides direct in vivo evidence of the progressive thinning in the visual cortex in patients with POAG. The most significant novel finding is that cortical thickness of the bilateral V5/MT+ areas decreases in the early stage of POAG. Thus, it should be paid more attention on early prevention of cortical degeneration associated with eye diseases, which might improve the clinical outcome for patients with POAG. Another notable finding is that the dorsal pathway may be more preferentially damaged than the ventral visual pathway in central visual system. Additionally, the cortical degeneration in V1and V2 is discrepant when the disease deteriorated from mild to severe stage. Finally, we showed that the thickness of visual cortex correlated well with RNFL thickness and visual field examination findings in POAG patients. Taken together, our findings suggest that the thickness of visual cortex is helpful in detecting POAG pathology and could serve as an indicator of disease severity.
